# Supplementary material for: Public health factors help explain cross country heterogeneity in excess death during the COVID19 pandemic
Source: Sci Rep. 2023 Sep 27;13:16196. doi: 10.1038/s41598-023-43407-0 (PMC10533501; doi:10.1038/s41598-023-43407-0)
Supplement: Supplementary file 1 — Supplementary Information. [file 41598_2023_43407_MOESM1_ESM.pdf]

Supplementary Material

Public Health Factors Help Explain Cross Country Heterogeneity in Excess Death During the COVID19 Pandemic

Min Woo Sun[1*†], David Troxell[2†] and Robert Tibshirani[1,2]

[1]Department of Biomedical Data Science, Stanford University, 450 Serra Mall, Stanford, 94305, CA, USA.
[2]Department of Statistics, Stanford University, 450 Serra Mall, Stanford, 94305, CA, USA.

*Corresponding author(s). E-mail(s): minwoos@stanford.edu;
Contributing authors: dtroxell@stanford.edu; tibs@stanford.edu;
†These authors contributed equally to this work.

Table S1. List of features for the initial dataset prior to any processing

Feature Name	Description	Intrinsic or Actionable
Days_Until_All_Vulnerable_Vacc_Elig	Days until the official government policy is that vulnerable groups are eligible for a COVID-19 vaccine	A
Percent_One_Dose_As_Of_Nov_1	Percent of individuals who have received at least one dose of a COVID-19 vaccine	A
Days_Until_Masks_Recommended	Number of days until the government's official policy became that masks are either recommended or required	A
Days_Until_Masks_Required	Number of days until the government's official policy became that masks are required in public spaces	A
Total_Days_Masks_Required_Public	Number of days in which the government's policy stated masks are required in public spaces	A
Total_Days_Masks_At_Least_Recommended	Number of days in which the government's policy stated masks are either recommended or required in public spaces	A
Days_Until_Workplace_Closures_Except_Key	Number of days until the government's official policy became that workplace closures are required for all citizens except for "key workers"	A
Total_Days_Workplace_Closures_Except_Key	Number of days in which the government's policy stated workplace closures are required for all citizens except for "key workers"	A
Total_Days_Workplace_Closures_Recommended	Number of days in which the government's policy stated workplace closures are either recommended for all, required for some groups, or required for all groups except for "key workers"	A
Total_Days_Stay_At_Home_Required_Except_Essentials	Number of days in which the government's policy stated citizens are required to not leave the house except for exercise, grocery shopping, and "essential" trips	A
Total_Days_Comprehensive_Contact_Tracing	Number of days in which contact tracing was performed for all cases	A

Days_Until_Income_Support	Number of days until the government's official policy became that at least some nonzero percentage of lost salary will be recovered to individuals who lost jobs or could not work	A
Total_Days_Income_Support	Number of days in which the government's policy was such that at least some nonzero percentage of lost salary will be recovered to individuals who lost jobs or could not work	A
Total_Freeze_Some_Financial_Obligations	Number of days in which the government's policy was that at least some percentage of financial obligation (loan payments, rent, utility bills, etc.) payments will be frozen	A
Days_Until_Freeze_Some_Financial_Obligations	Number of days until the government's official policy became that at least some percentage of financial obligation (loan payments, rent, utility bills, etc.) payments will be frozen	A
Days_Until_Testing_Key_Groups	Number of days until the government's official policy became that any citizen with symptoms or who met criteria such as key worker, back from travel, in hospital, contact with known case, etc. are eligible to receive a test	A
Total_Days_Open_Public_Testing	Number of days in which anyone (even asymptomatic) could receive a "drive through" test	A
Total_Days_Over_1_Test_Per_Thousand	Number of days in which over 1 test per thousand people was performed	A
Average_Tests_Per_Thousand_Per_Day	The daily number of tests performed on average in the study's timeframe	A
Urban_Pop_Percentage	Percent of citizens living in cities	I
Obese_Adult_Percentage	Percentage of adults who have a BMI $\geq$ 30, standardized by age	I
Hospital_Beds_Per_1000	Number of hospital beds per 1000 people	I
Nurses_And_Midwives_Per_1000	Number of nurses (and midwives as the definitions vary by country) per 1000 people	I
Percent_Using_Internet	Percent of people who use the internet	I
Population	Total population of country	I

People_Per_Sq_Km_of_Land	People per square kilometer of land in the country. Measures population density but excludes sq km of water	I
Adult_Literacy_Rate	Percent of literate people (ages 15 and above)	I
Percent_Ppl_Poor_Air_Quality	Percent of people exposed to levels of air pollution deemed hazardous by WHO	I
GDP_Per_Capita	GDP per capita (US $)	I
Health_Expenditure_Per_Capita	Total health expenditure per person	I
Percent_Health_Expenditure_Private	Percent of the total domestic health expenditure performed by private entities	I
Age_65_Older_Percent	Percent of citizens aged 65 and older	I
Ages_15_To_64_Percent	Percent of citizens aged 15 – 64	I
Ages_0_To_14_Percent	Percent of citizens aged 0 – 14	I

Table S2. List of all 80 countries that went into the analysis.

Country	ISO	Region	Sub-Region
Argentina	ARG	Americas	Latin America and the Caribbean
Australia	AUS	Oceania	Australia and New Zealand
Austria	AUT	Europe	Western Europe
Belgium	BEL	Europe	Western Europe
Benin	BEN	Africa	Sub-Saharan Africa
Burkina Faso	BFA	Africa	Sub-Saharan Africa
Bangladesh	BGD	Asia	Southern Asia
Bulgaria	BGR	Europe	Eastern Europe
Bolivia	BOL	Americas	Latin America and the Caribbean
Brazil	BRA	Americas	Latin America and the Caribbean
Canada	CAN	Americas	Northern America
Switzerland	CHE	Europe	Western Europe
Chile	CHL	Americas	Latin America and the Caribbean
Ivory Coast	CIV	Africa	Sub-Saharan Africa
Cameroon	CMR	Africa	Sub-Saharan Africa
Colombia	COL	Americas	Latin America and the Caribbean
Costa Rica	CRI	Americas	Latin America and the Caribbean
Czech Republic	CZE	Europe	Eastern Europe
Germany	DEU	Europe	Western Europe

Continued on next page

Country	ISO	Region	Sub-Region
Denmark	DNK	Europe	Northern Europe
Dominican Republic	DOM	Americas	Latin America and the Caribbean
Algeria	DZA	Africa	Northern Africa
Ecuador	ECU	Americas	Latin America and the Caribbean
Spain	ESP	Europe	Southern Europe
Ethiopia	ETH	Africa	Sub-Saharan Africa
Finland	FIN	Europe	Northern Europe
France	FRA	Europe	Western Europe
United Kingdom	GBR	Europe	Northern Europe
Ghana	GHA	Africa	Sub-Saharan Africa
Guinea	GIN	Africa	Sub-Saharan Africa
Greece	GRC	Europe	Southern Europe
Hungary	HUN	Europe	Eastern Europe
Indonesia	IDN	Asia	South-eastern Asia
India	IND	Asia	Southern Asia
Iran	IRN	Asia	Southern Asia
Iraq	IRQ	Asia	Western Asia
Israel	ISR	Asia	Western Asia
Italy	ITA	Europe	Southern Europe
Jordan	JOR	Asia	Western Asia
Japan	JPN	Asia	Eastern Asia
Kazakhstan	KAZ	Asia	Central Asia
Kenya	KEN	Africa	Sub-Saharan Africa
South Korea	KOR	Asia	Eastern Asia
Laos	LAO	Asia	South-eastern Asia
Lebanon	LBN	Asia	Western Asia
Sri Lanka	LKA	Asia	Southern Asia
Morocco	MAR	Africa	Northern Africa
Mexico	MEX	Americas	Latin America and the Caribbean
Mali	MLI	Africa	Sub-Saharan Africa
Myanmar	MMR	Asia	South-eastern Asia
Malaysia	MYS	Asia	South-eastern Asia
Nigeria	NGA	Africa	Sub-Saharan Africa
Nicaragua	NIC	Americas	Latin America and the Caribbean
Netherlands	NLD	Europe	Western Europe
Norway	NOR	Europe	Northern Europe
Nepal	NPL	Asia	Southern Asia
New Zealand	NZL	Oceania	Australia and New Zealand
Peru	PER	Americas	Latin America and the Caribbean
Philippines	PHL	Asia	South-eastern Asia
Poland	POL	Europe	Eastern Europe

Continued on next page

Country	ISO	Region	Sub-Region
Portugal	PRT	Europe	Southern Europe
Paraguay	PRY	Americas	Latin America and the Caribbean
Romania	ROU	Europe	Eastern Europe
Russia	RUS	Europe	Eastern Europe
El Salvador	SLV	Americas	Latin America and the Caribbean
Serbia	SRB	Europe	Southern Europe
Slovakia	SVK	Europe	Eastern Europe
Sweden	SWE	Europe	Northern Europe
Thailand	THA	Asia	South-eastern Asia
Tunisia	TUN	Africa	Northern Africa
Turkey	TUR	Asia	Western Asia
Tanzania	TZA	Africa	Sub-Saharan Africa
Uganda	UGA	Africa	Sub-Saharan Africa
Ukraine	UKR	Europe	Eastern Europe
United States	USA	Americas	Northern America
Uzbekistan	UZB	Asia	Central Asia
Venezuela	VEN	Americas	Latin America and the Caribbean
South Africa	ZAF	Africa	Sub-Saharan Africa
Zambia	ZMB	Africa	Sub-Saharan Africa
Zimbabwe	ZWE	Africa	Sub-Saharan Africa

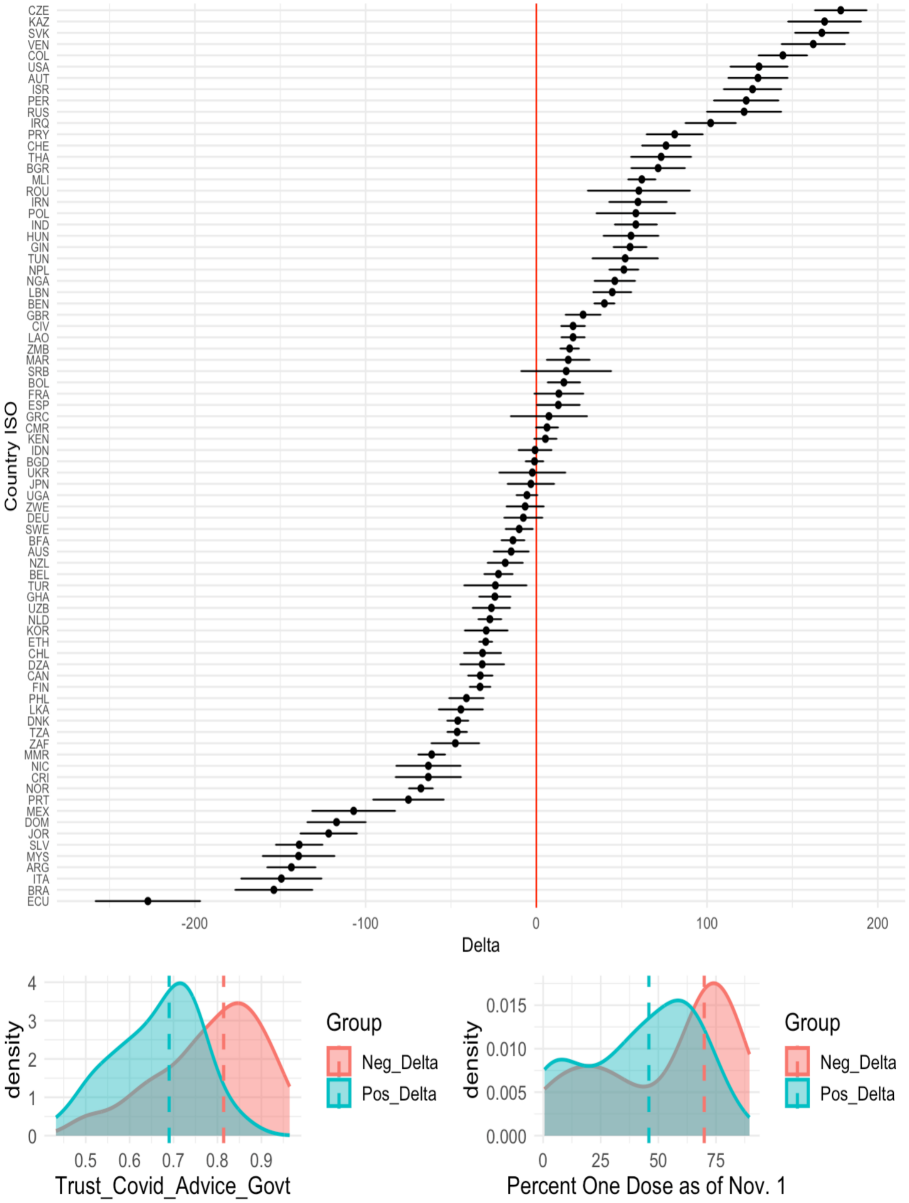

Fig. S1 Plot of all 80 countries' 95% bootstrap confidence intervals of the "delta value", which measures the difference in excess death prediction between the model fit on both intrinsic and actionable features, and the model fit only on intrinsic features.
